# Dislocation Strengthening without Ductility Trade-off in Metastable Austenitic Steels

**DOI:** 10.1038/srep35345

**Published:** 2016-10-14

**Authors:** Jiabin Liu, Yongbin Jin, Xiaoyang Fang, Chenxu Chen, Qiong Feng, Xiaowei Liu, Yuzeng Chen, Tao Suo, Feng Zhao, Tianlin Huang, Hongtao Wang, Xi Wang, Youtong Fang, Yujie Wei, Liang Meng, Jian Lu, Wei Yang

**Affiliations:** 1 State Key Laboratory for Strength and Vibration of Mechanical Structures, Xi’an Jiaotong University, Xi’an 710049, China; 2 College of Materials Science and Engineering, Zhejiang University, Hangzhou 310027, China; 3 Institute of Applied Mechanics, Zhejiang University, Hangzhou 310027, China; 4 Department of Mechanical and Biomedical Engineering, City University of Hong Kong, Hong Kong, China; 5 Northwestern Polytechnical University, Xi’an, 710072, China; 6 Center for High Pressure Science and Technology Advanced Research, Shanghai 201203, China; 7 College of Materials Science and Engineering, Chongqing, 400044, China; 8 School of Mechanical, Electronic and Control Engineering, Beijing Jiaotong University, Beijing 100044 China; 9 College of Electrical Engineering, Zhejiang University, Hangzhou 310027, China; 10 LNM, Institute of Mechanics, Chinese Academy of Sciences, Beijing 100190, China

## Abstract

Strength and ductility are mutually exclusive if they are manifested as consequence of the coupling between strengthening and toughening mechanisms. One notable example is dislocation strengthening in metals, which invariably leads to reduced ductility. However, this trend is averted in metastable austenitic steels. A one-step thermal mechanical treatment (TMT), *i.e.* hot rolling, can effectively enhance the yielding strength of the metastable austenitic steel from 322 ± 18 MPa to 675 ± 15 MPa, while retaining both the formability and hardenability. It is noted that no boundaries are introduced in the optimized TMT process and all strengthening effect originates from dislocations with inherited thermal stability. The success of this method relies on the decoupled strengthening and toughening mechanisms in metastable austenitic steels, in which yield strength is controlled by initial dislocation density while ductility is retained by the capability to nucleate new dislocations to carry plastic deformation. Especially, the simplicity in processing enables scaling and industrial applications to meet the challenging requirements of emissions reduction. On the other hand, the complexity in the underlying mechanism of dislocation strengthening in this case may shed light on a different route of material strengthening by stimulating dislocation activities, rather than impeding motion of dislocations.

Dislocations are major plastic deformation carriers in most metals. Traditional strengthening methods involves the controlled creation of internal defects and boundaries, such as solution atoms and grain/phase boundaries, so as to impede motion of dislocations^1^,^2^,^3^,^4^,^5^. Unfortunately, the strength enhancement is generally compromised by ductility reduction, known as the strength-ductility trade-off dilemma in material science^6^. One solution to this trade-off relationship lies in a strengthening strategy that not only obstructs dislocation motion, but also provides extra dislocation storage capability. Unlike grain boundaries, nanoscale coherent twin boundaries (CTBs) can accommodate dislocations whose density is orders of magnitude higher than that stored in deformed nanocrystalline metals^1^,^7^,^8^,^9^. The uniform elongation can increase monotonically with the decrease of twin spacing in copper, as a reflection of the increasing CTB-facilitated dislocation storage^9^. Furthermore, dedicated hierarchy structures have been created in metals by mechanical treatment to balance the strength and ductility as well. The most successful examples include introducing three levels of hierarchical nanotwins, producing gradient nano grained or twinned structures, and generating gradient grains with embedded nanotwins^4^,^5^. Aside from obstructing dislocation motion, the dislocation density can be continuously pumped up by orders of magnitude by deformation as a consequence of the strain or stress-induced phase transformation^10^,^11^,^12^. This conception has led to the invention of transformation-induced plasticity (TRIP) steels, possessing a combination of exceptional ultimate strength, formability and hardenability. Though the ultimate strength of TRIP steels can reach 1 GPa, the yielding strength is generally as low as ~300 MPa in annealed states^13^. Various strategies, such as grain refinement and cold working, have been employed to increase the yielding strength, inevitably sacrificing ductility to some degree^14^,^15^,^16^,^17^,^18^. In this work, we report that a one-step thermal mechanical treatment (TMT), *i.e.* hot rolling, can effectively enhance the yielding strength of the metastable austenitic steel (a type of TRIP steel) from 322 ± 18 MPa to 675 ± 15 MPa, while retaining both the formability and hardenability. It is noted that no boundaries are introduced in the optimized TMT process and all strengthening effect originates from dislocations with inherited thermal stability. Especially, the simplicity in processing enables scaling and automotive industrial applications to meet the challenging requirements of emissions reduction. On the other hand, the complexity in the underlying mechanism of dislocation strengthening in this case may shed light on a different route of material strengthening by stimulating dislocation activities, rather than impeding motion of dislocations.

## Results

### Microstructural characterization and tensile behaviors of TMT metastable austenitic steel

**Figure 1:**
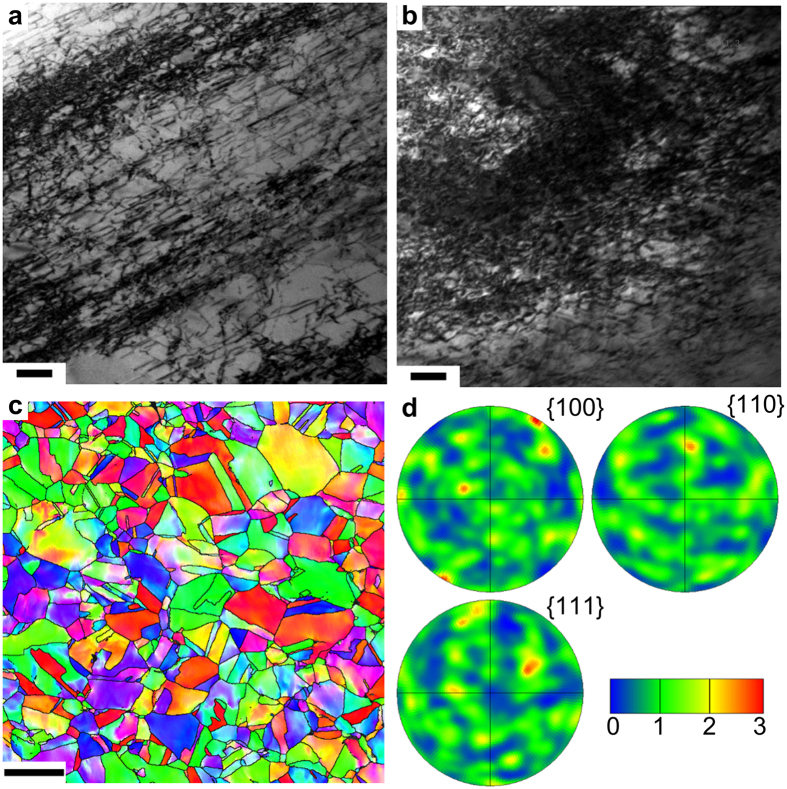
Microstructural characterization of warm-rolled metastable austenitic steels. Two-beam bright field TEM images of TMT samples with (**a**) 10% and (**b**) 20% thickness reduction. Zone axis is (110) and***g*** = (111). Scale bar: 100 nm. (**c**) (EBSD) image of the TMT sample with 20% thickness reduction. Scale bar: 100 um. (**d**) The responding pole figure of (**c**) showing weak texture characteristics.

The metastable austenitic steel is used in our study. The detailed characterization and processing is given in the Methods section. The initial material has an average grain size of 40 um with equal-axed shape and is nearly free of defects (Supplementary Fig. S1). The stacking fault energy at room temperature (RT) is in the range of 5–15 mJ/m^2^ in the austenitic structure^19^. The RT deformation is characterized by the sequential presence of stacking faults, ε-martensites and α-martensites. Such TRIP steel has a typical yielding strength of ~300 MPa but superior hardening capacity, resulting uniform elongation and high ultimate strength. Hot rolling is an effective way in injecting dislocations into metastable austenitic steels while suppressing the phase transformation. Meanwhile, the elevated temperature raises the stacking fault energy and narrows the stacking fault ribbons, facilitating the cross-slip process. As compared in Fig. 1a,b, the dislocation density increases with the amount of reduction in thickness. Constriction of the stacking fault ribbon is retained at room temperature due to the interactions among the forest dislocations. Only a small portion can be observed to dissociate on the slip planes. Under such moderate deformation, no obvious cellular substructure has been identified. It is noted that small amount of ε-martensite forms beyond a critical rolling reduction that depends on the temperature. The maximum dislocation density has been achieved at the highest rolling temperature (450 °C) without sensitization and the corresponding critical rolling reduction (20%). The TMT effect on the grain structure has been investigated by the electron backscattering diffraction (EBSD) (Fig. 1c). The grains keep the equi-axed shape with no substantial difference in aspect ratio and GBs are slightly curved. The pole figure reveals no strong texture developed in the austenitic steels (Fig. 1d).

**Figure 2:**
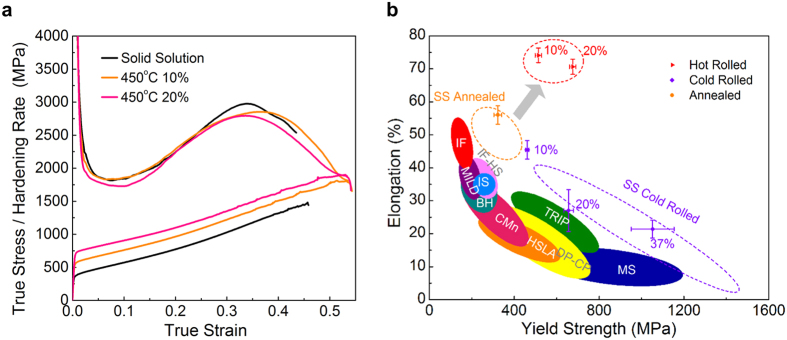
Strengthening of metastable austenitic steels by TMT treatment. (**a**) Stress-strain curves for the TMT samples. (**b**) Comparison of the uniform elongation versus yield strength for typical steels.

The stress–strain curves shown in Fig. 2a indicate that the thermal mechanical treatments have led to substantial increases in yielding strength, but no adverse effect on the ductility. Serration flow is observed in all testing samples at larger tensile strains, revealing competition between the increase in tensile stress and stress relaxation induced by phase transformation. The strain hardening rate retains the typical up-turn form and the same magnitude, implying the least disturbance of TMT to the TRIP mechanism. Figure 2b summarizes the tensile testing results. After TMT, the 0.2% yielding strength increases from 322 ± 18 MPa to 513 ± 15 MPa and 675 ± 15 MPa for samples with 10% and 20% reduction in thickness, respectively. Meanwhile, the uniform elongation increases from 54% ± 3% to 74% ± 2% and 71% ± 2%. The simultaneous enhancement presents a striking contrast to the usual strength–ductility trade-off in other steels, as well as the cold-worked samples.

### Interactions between dislocation and phase transformation

**Figure 3:**
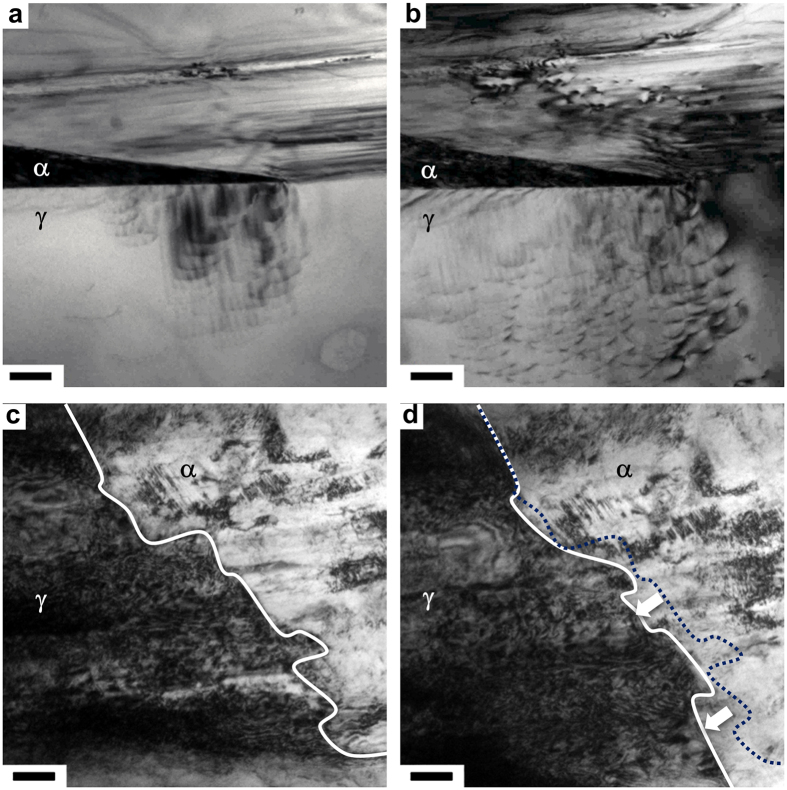
Phase boundary as effective dislocation source. (**a,b**) TEM image sequence of an *in-situ* deformed solid solution treated metastable austenitic steel. The electron beam is along the [011]α. Scale bar: 200 nm. (**c,d**) TEM image sequence of an *in-situ* deformed TMT samples with 20% thickness reduction. The phase boundary are marked by the solid lines. The dashed line in (**d**) locates the original phase boundary in (**c**). The direction of the step-wise propagation is indicated by the arrows in (**d**). Scale bar: 200 nm.

To understand the mechanism of plastic deformation in the metastable austenitic steel, we found it particularly insightful to observe the interactions between dislocations and phase transformation by *in-situ* transmission electron microscopy (TEM). The experimental details are provided in the Methods section. Under *in-situ* deformation, wedge-like α-martensites are frequently observed in solid-solution-treated samples (Supplementary Fig. S2). The burst nucleation and growth processes can hardly be captured due to the limited temporal resolution of the current setup. However, the stepwise displacement loading mode allows careful observation of the dislocation emission from phase boundaries, as recorded in Supplementary Movie S1. The BCC nuclei have the Kurdjumov-Sachs orientation relationship to the FCC matrix, *i.e.* [111]_BCC_//[101]_FCC_ and (110)_BCC_//(111)_FCC_. In this particular setup, the electron beam is along [101]_BCC_ and slightly off [−233]_FCC_ (Supplementary Fig. S3) and all four {111}_FCC_ slip planes are inclined to the view direction. Consequently, dislocation activity in the FCC matrix can be clearly identified. Large amount of dislocations are pumped into the FCC matrix from the phase boundary during straining, as shown in two consecutive snapshots (Fig. 3a,b), even when the local stress is not high enough to advance the phase boundary. Two active slip planes can be identified, as marked as marked by letters A and B in Supplementary Fig. S3. Clearly, dislocations are preferentially nucleated around the tip region. Further increase of the load activates the phase boundary far from the tip as active dislocation sources (Fig. 3b). It is noted that the phase boundary coherency deteriorates with dislocation generation (Fig. 3b). In TMT samples, the α-martensites have irregular geometries with abrupt steps located on phase boundaries (Fig. 3e,f). Supplementary Movie S2 reveals that the front advances into the FCC matrix with high-density dislocations in a piecewise style. Given the local stress as the direct driving force, this special growth mode is a direct consequence of the back stress non-uniformity induced by pre-stored forest dislocations. Dislocation lines can hardly be identified due to the complex tangled structure. Dislocation activity is estimated from the fast and continuous change in contrast as a reflection of the local strain field variation due to dislocation motion. Clearly, dislocations are more active at the phase boundary, especially near the sharp corners connecting boundary steps. The stress singularity is closely related to the abrupt change in elastic moduli and geometries. The terrace-like phase boundaries in TMT samples serve as more effective dislocation sources. The *in-situ* investigation suggests that the pre-stored high density dislocation does not pin the phase boundaries, but promotes phase transformation. In contrast to frequent dislocation movements in the FCC matrix, the slips in α-martensite are initiated only occasionally, indicating that the martensite is much harder than the austenite.

**Figure 4:**
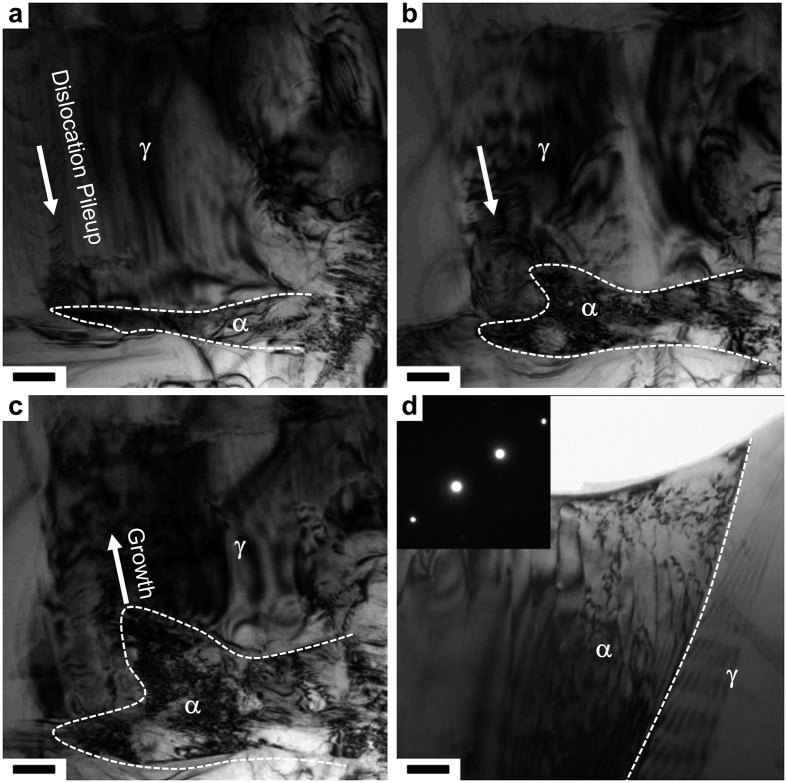
Dislocation pileup against the phase boundary promotes α-martensite transformation. (**a–c**) TEM image sequence of an *in-situ* deformed metastable austenitic steel reveals the irregular growth of α-martensite. The dashed lines delineate the phase boundary. The arrows in (**a,b**) point to the direction of dislocation movements. The dislocation array piles up against the phase boundary. The preferential α-martensite growth is indicated by the arrow in (**c**). Scale bar: 100 nm. (**d**) The two-beam bright field TEM image shows typical dislocation arrangements in an α-martensite. Inset is the corresponding SAED pattern from the α-martensite with *g* = (01–1). Scale bar: 100 nm.

It is intriguing to study the key role of dislocations in phase transformation by observing dislocation pileups against the γ/α interface. An example is given in Supplementary Movie S3. Clearly, the phase boundary strengthening results from impediment to dislocation motion (Fig. 4a), similar to the role of GBs. In general, stress concentration appears at the dislocation-GB intersection and increases with the number of pileup dislocations. The local stress relaxation is always associated with GB decohesion or dislocation nucleation in neighboring grains. Different scenario has been observed for phase boundaries, as shown in Fig. 4b,c. The α-martensite preferentially grows against the pileup direction. It is not clear whether dislocations in austenite can be inherited by α-martensite or simply disappear after phase transformation. The sessile nature of dislocations in BCC lattice can no longer serve as a stress concentrator. In this way, the upper bound of stress is effectively limited, which is lower than the γ/α interfacial strength. Figure 4d shows the typical dislocation morphology in α-martensite, no matter the parent phase is solid solution or hot rolled. It is generated during the phase transformation process, instead of deforming the BCC lattice. The dislocations in the α-martensite appear to belong to two different slip planes (Supplementary Fig. S4). Tilting to a series two-beam conditions confirm that the dislocations are of the type <111>/{211}, which are glissile screw dislocations in BCC lattice. Edge dislocations are identified in HRTEM images of martensite nucleated in the severely deformed austenite. In dozens of *in-situ* TEM observations, dislocations in α-martensites are rather immobile, keeping a nearly constant dislocation density. It is also likely that these small plates have inherited fine dislocation substructures from the deformed metastable austenite, leading to a harder martensites.

### Dispersive martensite distribution

**Figure 5:**
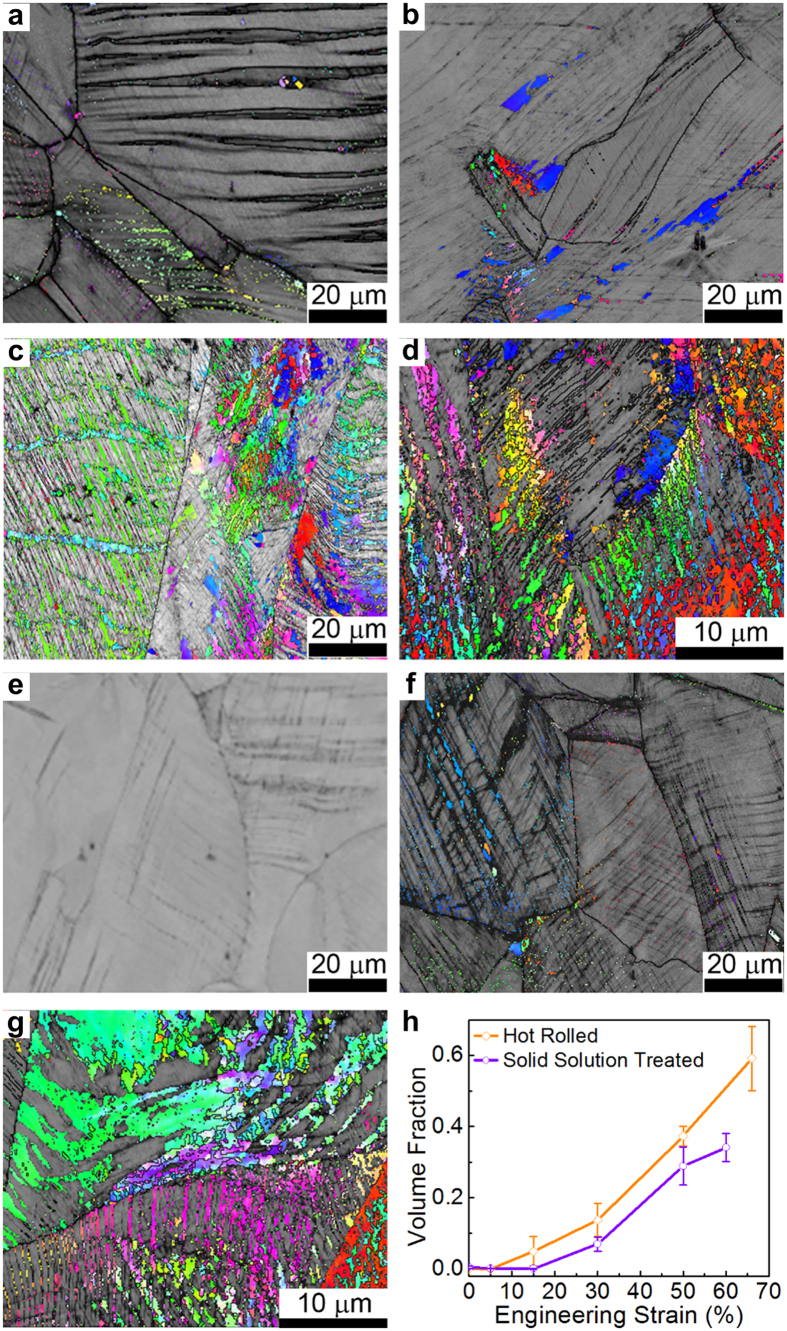
Microstructural evolution under tension. EBSD images of warm rolled samples with tensile strain of (**a**) 15%, (**b**) 30%, (**c**) 50% and (**d**) 67%. All TMT samples have the same rolling reduction in thickness of 20%. EBSD images of solid solution treated samples with tensile strain of (**e**) 15%, (**f**) 30% and (**g**) 60%. (**h**) The XRD-measured volume fraction α-martensite as a function of tensile strain for both TMT and solid solution treated samples. The rolling reduction in thickness of TMT samples is 20%. The tensile tests were performed at strain rates 1.0×10^−3^ s^−1^ and room temperature. Supplementary Fig. S6 shows the corresponding stress-strain curves.

The α-martensite distribution is another key factor in shaping the ductility. Figure 5 (a–e) show that α-martensites preferentially nucleate near the intersection of slip zones and the growth is largely bounded by the strip-like severely deformed regions in the interior of austenite grains. For the relatively uniform distribution of slip zones, the coalescence of α-martensites located in neighboring slip zones is greatly suppressed. Further TEM characterization of the fractured samples reveals that the α-martensite grains have the same orientation and are aligned in strips with sub-micron sizes and non-equal-axed shapes, separated by austenite phase (Supplementary Fig. S5). The dispersive distribution of α-martensites of sub-micron size suggests abundant dislocation sources inside the metastable austenitic stainless steels, rendering high ductility of the metastable austenitic steels. Figure 5 directly compares the α-martensite distribution between the TMT and solid solution treated samples under various tensile strains. The characteristics of both types of samples are similar at larger strains, even though α-martensites nucleate at a much earlier stage of deformation in TMT samples. The volume fraction of α-martensites in both TMT and solid solution treated samples is plotted as a function of plastic strain in Fig. 5h. The only notable structural difference of two types of samples is the high density of initial dislocations in the TMT samples. This result clearly demonstrates that the pre-existed dislocations promote α-martensite phase transformation.

## Discussion

Strength and ductility are mutually exclusive if they are manifested as a result of the coupling between strengthening and toughening mechanisms. Increase of yield strength demands restriction to dislocation motion that carries major plastic deformation. The dislocation mobility, on the other hand, is closely correlated to the ductility of metals. One notable example is dislocation strengthening in metals, which invariably leads to reduced ductility. A counterintuitive result has been achieved in nano-twinned copper, which shows simultaneous enhancement of strength and ductility with decreasing twin-boundary spacing^9^. A transition from strengthening to softening is observed around a critical twin thickness ~20 nm, while the plasticity monotonically increases with twin density. In nano-twinned copper, TBs are barriers for dislocation motion while the intersections between TBs and GBs serve as effective dislocation sources. The two microscopic processes are spatially separated and physically linked by TBs, leading to the abnormal strength-ductility relation^8^. It is also possible to alter the tradeoff if strengthening and toughening mechanisms can be decoupled, *i.e.* yield strength is controlled by initial defects while ductility relies on the capability of the material to nucleate new defects to carry plastic deformation.

Several factors may contribute to yield strength of metastable austenitic steels. However, the preexisting dislocations in TMT samples are likely to dictate. Dispersion of alloy carbides or martensites has not been observed by careful TEM and XRD study, excluding strengthening mechanisms from second phases. The yield strength of both cold and hot rolled samples is comparable at the same rolling reduction, albeit the presence of martensites during cold rolling. The effectiveness of hot rolling in dislocation generation can be estimated by Orowan equation^20^:


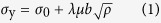


where *σ*_y_ and *σ*_0_ are the yield stresses with or without initial dislocations, *μ* the shear modulus, *b* the Burger’s vector, *ρ* the dislocation density and *λ* the numerical constant in a range of 0.3 to 0.6 for different FCC metals. The dislocation density is ~10^15^ m^−2^ for the hot-rolled sample with 20% thickness reduction, which is about one order of magnitude lower than that of severely plastically deformed metals. For none phase transformable metals, such as copper or aluminum, further tensile deformation drives the tangled dislocation network into a cellular substructure, affecting both dislocation nucleation and storage capability. The initial strengthening mechanism intervenes in the deformation path by occupying the room for further deformation. In metastable austenitic steels, martensitic transformation is an essential part of the plastic deformation process, as it substantially increases the dislocation density. Our *in-situ* TEM study reveals little effect of dislocations on phase transformation. Only an insignificant portion of atoms in the cores is dislocated from FCC lattice even with a dislocation density of 10^15^ m^−2^. The associated lattice distortion is negligible as compared to the atomic displacement during martensite transformations. In contrast, martensite growth terminates at high-angle GBs or TBs due to its dependence on coherency with the surrounding austenite. Furthermore, terraced phase boundaries, caused by submicron-scale stress field inhomogeneity, are more effective in dislocation multiplication. Early theory also proposes the possibility that the martensite barrier can be reduced by the help of the elastic strain energy, as well as dissociation of the preexisted dislocations^21^. This is evident by comparing the microstructural evolution between TMT and solid solution samples at same tensile strains (Fig. 5): (1) the TMT process leads to phase transformation at earlier deformation stage; and (2) α-martensite content is higher in TMT samples at the same tensile strain. It is also noted that the average size and the dispersity of α-martensites are comparable in both TMT and solid solution samples. Each deformed austenite grain is divided into more separated regions by increasing number of martensites, which are much smaller than those in conventional heat treatments. The typical microstructure of TMT samples right before fracture reveals an intercalation of α-martensite and austenite with the lamella spacing ~500 nm (Supplementary Fig. S6). The fine-scale martensites greatly raise the strain-hardening capacity and the uniform distribution effectively suppresses strain localization. Both contribute to the excellent ductility in metastable austenitic steels strengthened by high density dislocations. The strength-ductility tradeoff is not observed in this case. The success of this method relies on the decoupled strengthening and toughening mechanisms in metastable austenitic steels, in which yield strength is controlled by initial dislocation density while ductility is retained by the capability to nucleate new dislocations to carry plastic deformation. Following this guideline, other methods may be developed to enhance material performance in metal manufacturing.

## Methods

### Material preparation

The commercial steels AISI 301 (EN1.4310) were supplied by Outokumpu, Finland. The composition is given in Supplementary Table S1 in the online supporting materials. The initial materials were cut from 1 mm-thick sheets, solid solution treated at 1050 °C for 2 h with Ar ambient and then quickly quenched into water. The hot rolling was performed at 450 °C.

### Mechanical testing

The dimension of tensile specimens is 6.0 mm in width and 25.0 mm in gage length. The thickness is 1.0 mm, 0.9 mm and 0.8 mm for samples after solid solution treatment, rolling reduction in thickness of 10% and 20%, respectively. More detailed geometries can be found in Supplementary Fig. S7. Tensile tests were carried out at room temperature using a universal testing machine (MTS Alliance RT/30) at a strain rate of 1×10^−3^ s^−1^. At least three samples were tested to confirm the repeatability. For the TMT samples, the tensile axis is parallel to the rolling direction.

### TEM characterization

*In-situ* TEM experiments were carried out with a straining holder (Gatan 654) equipped in a JEM-2100 TEM operated at 200 kV. The specimens were strained by controlling the total elongation *via* a step motor in the straining holder. TEM samples were thinned by twin-jet electropolishing in the solution containing 10 vol.% perchloric acid and 90 vol.% acetic acid at 10 °C.

Dislocations were analyzed by tilting samples to various two-beam conditions with ***g*** = (110), (101) *etc*. Burgers vectors were determined by employing the ***g***·***b*** criterion. The glide planes were resolved by combining bright-field TEM images and corresponding SAED patterns.

### X-Ray diffraction and EBSD characterization

Samples for both XRD and EBSD tests were firstly mechanical ground with abrasive papers to remove the surface defect and followed by electrochemical polishing to avoid artifacts induced by grinding. The XRD measurements were conducted in a Bruker D8 Discover X-ray diffractometer with a Co-Kα radiation. The incident X-ray beam was collimated to a spot with a diameter of 0.5 mm on the surface of specimens. A Bruker VÅNTEC-500 two-dimensional detector was used to record the diffraction patterns. Supplementary Fig. S8 gives the setup of X-Ray beam alignment and sample position. To minimize the effect of texture, the measurements were carried out at Ψ = 0°, 25°, and 50°, respectively. The volume fractions of different phases at each Ψ angle were determined by analyzing the measured XRD patterns quantitatively using the Brucker TOPAS program. The mean volume fractions were obtained by averaging the volume fractions obtained at different Ψ angles.

EBSD characterization is performed in a FEI Quanta 250 FEG-SEM equipped with the Oxford EBSD detector and HKL channel 5 OIM software. The indexing quality, in terms of confidence index, is higher than 92% for all scans.

## Additional Information

**How to cite this article**: Liu, J. *et al*. Dislocation Strengthening without Ductility Trade-off in Metastable Austenitic Steels. *Sci. Rep.* **6**, 35345; doi: 10.1038/srep35345 (2016).

## Supplementary Material

Supplementary Information

Supplementary Movie S1

Supplementary Movie S2

Supplementary Movie S3
