# Corrigendum: Dislocation Strengthening without Ductility Trade-off in Metastable Austenitic Steels

**DOI:** 10.1038/srep46938

**Published:** 2018-01-29

**Authors:** Jiabin Liu, Yongbin Jin, Xiaoyang Fang, Chenxu Chen, Qiong Feng, Xiaowei Liu, Yuzeng Chen, Tao Suo, Feng Zhao, Tianlin Huang, Hongtao Wang, Xi Wang, Youtong Fang, Yujie Wei, Liang Meng, Jian Lu, Wei Yang

Scientific Reports 6: Article number: 35345; 10.1038/srep35345 published online: 10 14 2016; updated: 01 29 2018.

The original version of this Article contained errors in the affiliation list. Affiliations 1, 2 and 3 were incorrectly listed as affiliations 2, 3 and 1 respectively.

In addition, Jiabin Liu was incorrectly affiliated with ‘State Key Laboratory for Strength and Vibration of Mechanical Structures, Xi’an Jiaotong University, Xi’an 710049’. The correct affiliation for Jiabin Liu is listed below:

College of Materials Science and Engineering, Zhejiang University, Hangzhou 310027, China

Finally, an additional affiliation for Xiaoyang Fang was omitted. The correct affiliations for Xiaoyang Fang are listed below:

College of Materials Science and Engineering, Zhejiang University, Hangzhou 310027, China

State Key Laboratory for Strength and Vibration of Mechanical Structures, Xi’an Jiaotong University, Xi’an 710049, China

These errors have now been corrected in the HTML and PDF versions of the Article.

