# Supplementary material for: Dislocation Strengthening without Ductility Trade-off in Metastable Austenitic Steels
Source: Sci Rep. 2016 Oct 14;6:35345. doi: 10.1038/srep35345 (PMC5064382; doi:10.1038/srep35345)
Supplement: Supplementary Information [file srep35345-s1.pdf]

Dislocation Strengthening without Ductility Trade-off in Metastable Austenitic Steels

Supplementary video legends

Video S1. Dislocation emission from phase boundaries.

Video S2. The phase front advances into the FCC matrix with high-density dislocations.

Video S3. Dislocation pileups against the α/γ interface leads to phase transformation.

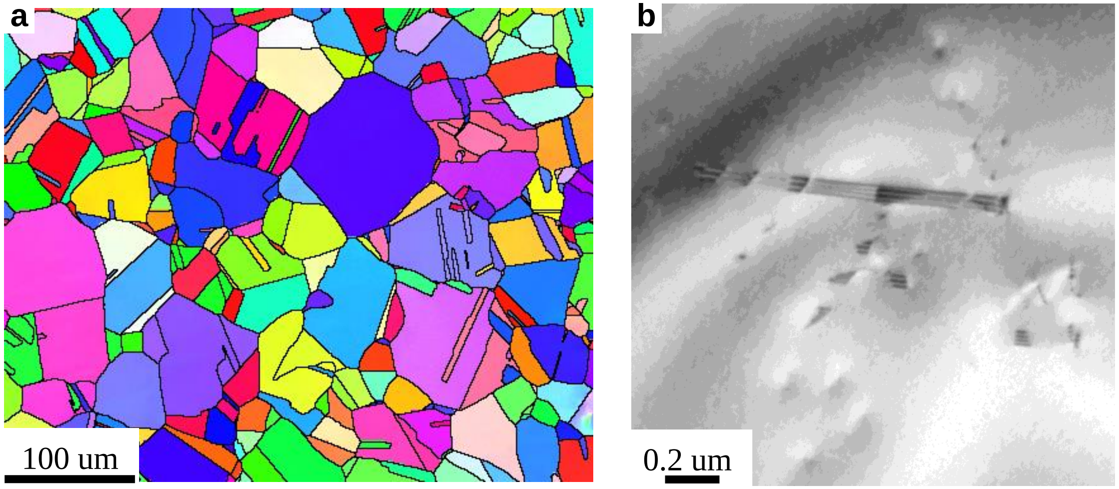

Figure S1 | Microstructural characterization of solid solution treated metastable austenitic steels. (a) Electron backscattering diffraction (EBSD) image; (b) Bright field TEM image of the interior of a grain.

Table S1. Chemical compositions of the metastable austenitic steels (wt.%). The balanced composition is Fe.

C	Si	Mn	Cr	Ni	Mo	N
0.14	0.52	1.76	16.61	6.02	0.05	0.18

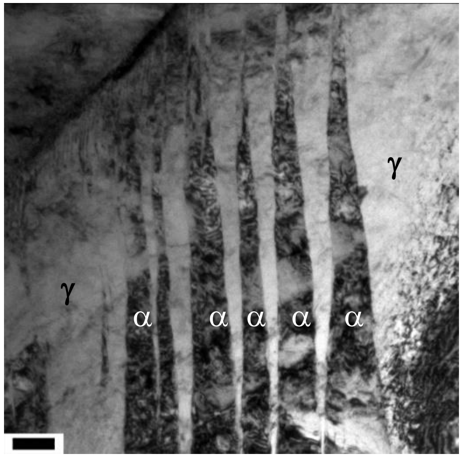

Figure S2 | Wedge-like α-martensites are frequently observed in solid-solution-treated samples under *in-situ* deformation. The Greek letters α and γ indicate the nucleated α-martensite and the austenite matrix. Scale bar: 200 nm.

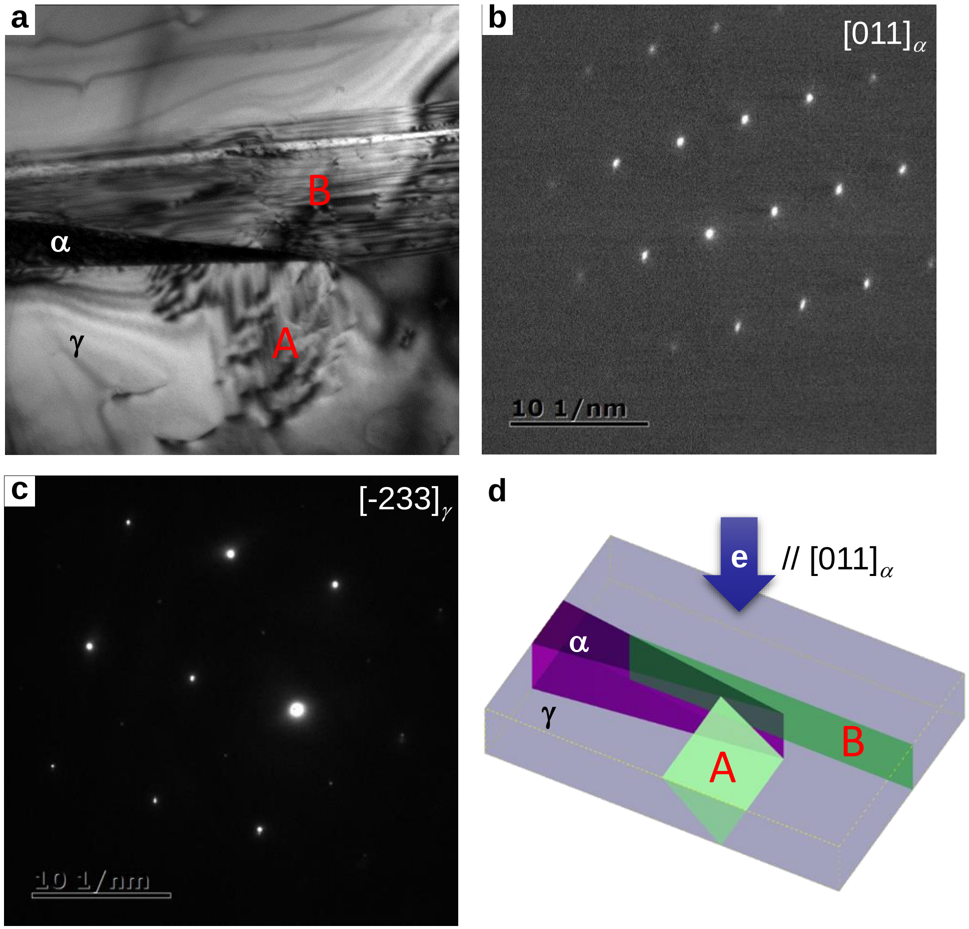

Figure S3 | Sample setup for the *in-situ* deformation. (a) TEM image of nucleated α-martensite during *in-situ* deformation. The letter A and B indicate two different active slip planes in the γ phase. (b) Selected area electron diffraction (SAED) pattern of the α-martensite shows that the zone axis is $[011]_{\alpha}$. (c) SAED pattern of the austenite matrix (γ phase) shows that the electron beam is slightly off $[-233]_{\gamma}$. Based on the diffraction, the relative orientation between the α- and γ-phases is equivalent to the Kurdjumov-Sachs orientation relationship, *i.e.* $[111]_{\alpha}$ // $[101]_{\gamma}$ and $(110)_{\alpha}$ // $(111)_{\gamma}$. (d) A schematic setup of the *in-situ* TEM sample. The two active slip planes (A and B) can be identified to be (111) and (-111), respectively.

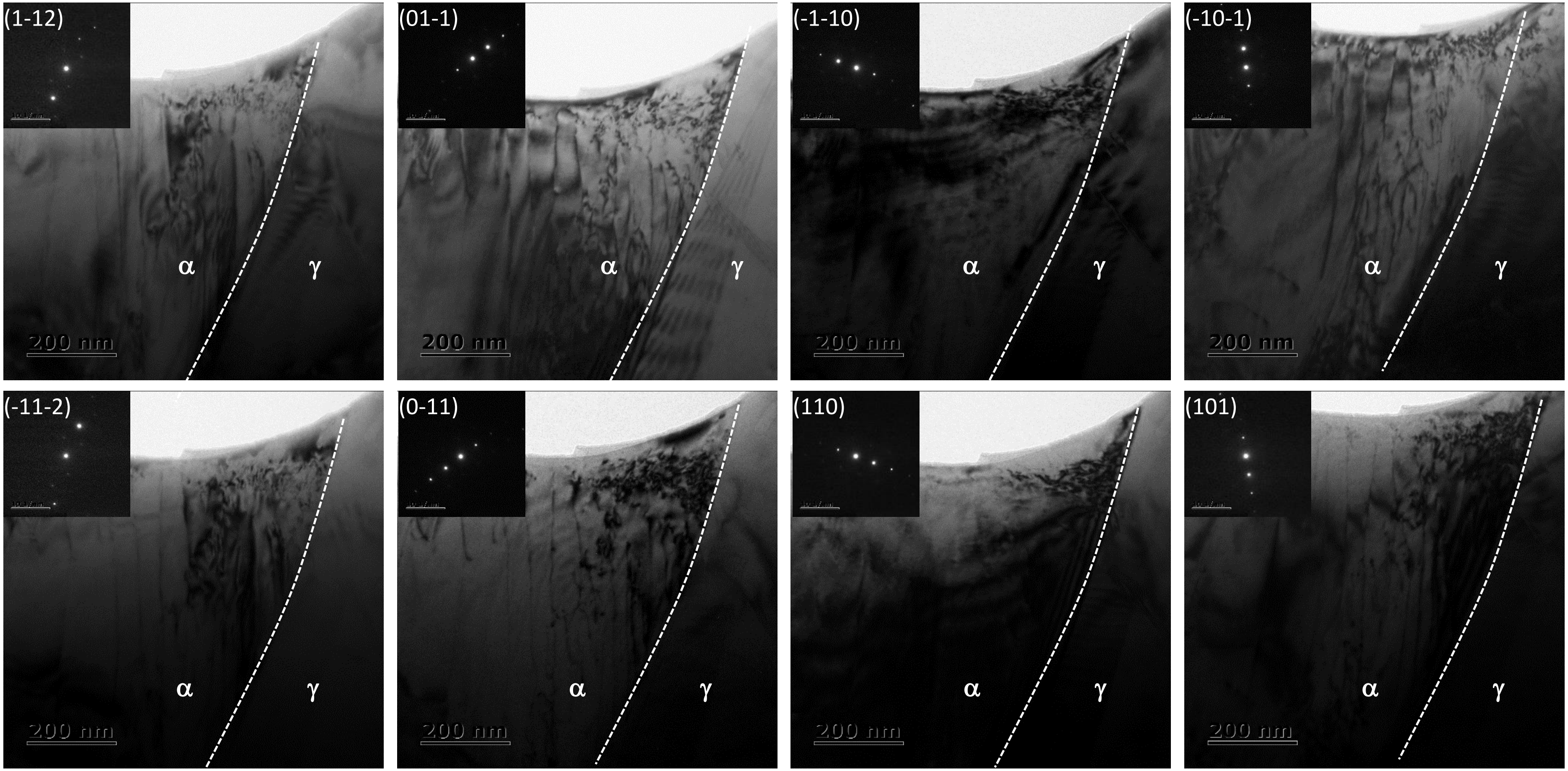

g	(1-12)	(-11-2)	(01-1)	(0-11)	(-1-10)	(110)	(-10-1)	(101)
	Visible	Visible	Visible	Visible	Invisible	Invisible	Visible	Visible

Figure S4 | Characterization of dislocations inside α-martensite. The sample was tilted to a series of two-beam conditions with g indicated inside the inset SAED patterns of the α-martensite. The dashed line indicates the phase boundary. The visibility of the dislocations is summarized in the table. Based on the results, it can be identified that the Burgers vector ***b* = ± [1-11]/2.**

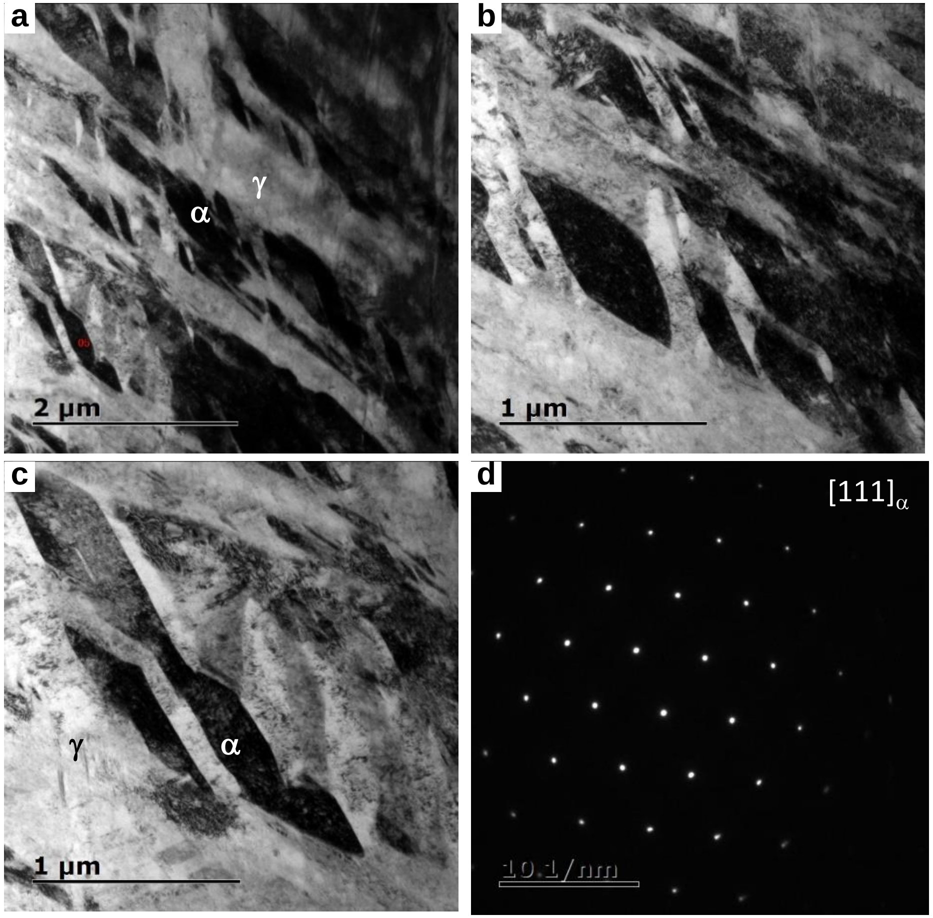

Figure S5 | Characterization of α-martensite in a TMT sample at tensile strain of 50%. (a-c) Bright field TEM images showing the morphologies and distribution of α-martensites in the austenite matrix. The electron beam is accurately aligned to the $[111]_{\alpha}$. (d) SAED pattern of the α phase. The zone axis is $[111]_{\alpha}$.

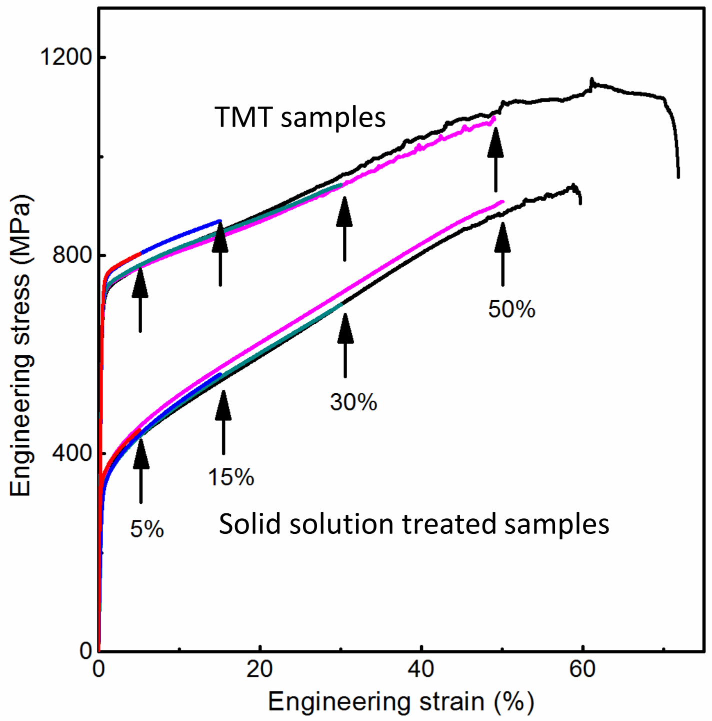

Figure S6 | The stress-strain curves of samples for *ex-situ* characterization. The samples were used for *ex-situ* XRD, EBSD and TEM characterization of the corresponding microstructures at different tensile strains. For each type of samples, the stress-strain curves nearly coincide with each other, implying the similar microstructural evolution.

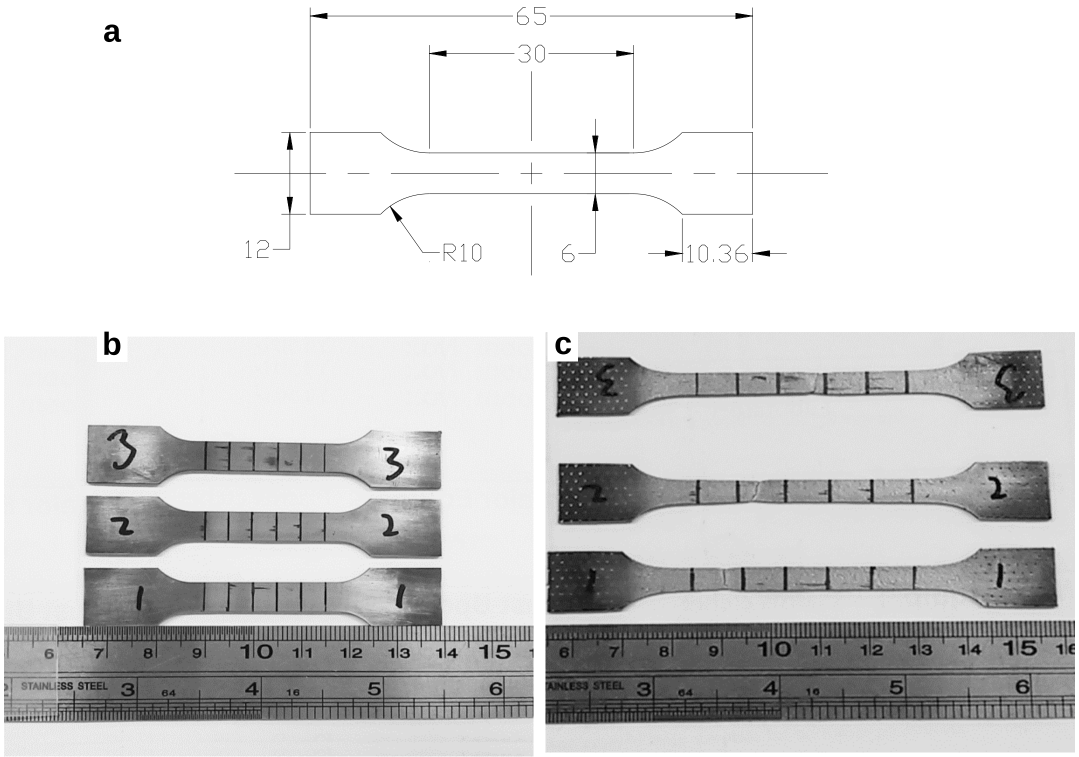

Figure S7 | Characterization of the tensile testing specimens. (a) Autocad drawing. Optical photos of a batch of samples (b) before and (c) after tensile tests.

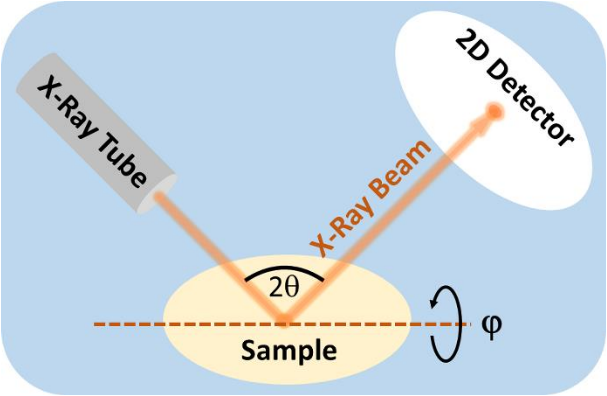

Figure S8 | Schematic diagram showing the X-Ray diffraction setup for this study. The 2θ and ϕ axes are indicated.
